# Effects of Au Nanoparticles Suspended in Chlorobenzene Antisolvent on Mixed-Halide Perovskites

**DOI:** 10.1021/acsomega.5c05967

**Published:** 2025-09-16

**Authors:** Eduardo H. dos Santos Rosa, Andreia de Morais, Francineide Lopes de Araújo, Paul Zimmermann, Alexander Hinderhofer, Jilian Nei de Freitas, Rafael Eleodoro de Góes, Arandi Ginane Bezerra, Andreia Gerniski Macedo, Wilson José da Silva, Ana Flávia Nogueira, Frank Schreiber

**Affiliations:** † CPGEI, Department of Electronics, 74354 Universidade Tecnológica Federal do Paraná, 80230-901 Curitiba, PR, Brazil; ‡ CTI Renato Archer, 13069-901 Campinas, SP, Brazil; § Laboratório de Nanotecnologia e Energia Solar (LNES), Institute of Chemistry, Universidade Estadual de Campinas (UNICAMP), 13083-970 Campinas, SP, Brazil; ∥ Institute for Applied Physics, University of Tübingen, 72074 Tübingen, Germany; ⊥ PPGFA, Department of Physics, Universidade Tecnológica Federal do Paraná, 80230-901 Curitiba, PR, Brazil

## Abstract

In this work, Au nanoparticles (NPs) were synthesized by laser ablation in liquids (LASiS) by using chlorobenzene, resulting in a stable suspension with a plasmon resonance band around 560 nm. This Au NPs suspension was subsequently used in the antisolvent step for the preparation of the CsFAMA perovskite films. Morphological analyses revealed an increase in the grain size in the Au NPs-modified films, attributed to Au NPs-assisted heterogeneous nucleation. In situ GIWAXS measurements were conducted during film crystallization, pointing out that in the Au NPs-modified films prepared with diluted suspension, the peaks corresponding to the cubic α-phase formed faster and with reduced PbI_2_ content, when compared to the control film produced without Au NPs. The characterization of solar cell devices fabricated with Au NPs-modified CsFAMA films presented the influence of the NPs concentration on photovoltaic performance. Devices prepared with diluted Au NPs suspensions exhibited a higher power conversion efficiency (PCE) over time, improved stability, and a reduced hysteresis index.

## Graphical abstract

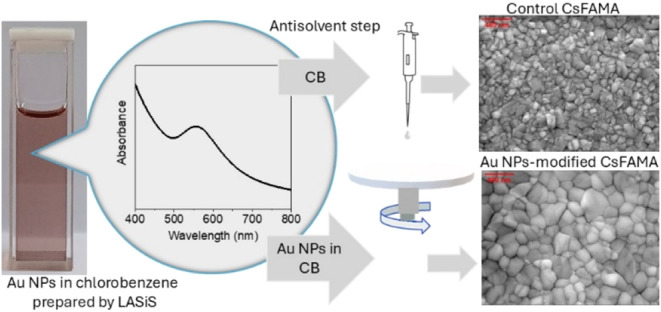

## 1 Introduction

Perovskite solar cells (PSCs) have presented significant advances in terms of energy conversion, reaching values of power conversion efficiency (PCE) in the order of 26.7%.^1^ Recent research has focused on increasing the PCE and outdoor stability for future commercialization. In this context, the degree of crystallization of the perovskite (PVK) has been identified as a feature that contributes to improving both PCE and stability parameters.^2–4^

In terms of morphology, smaller-sized PVK grains have reduced the stability of PSC due to larger surface area and grain boundaries in these films. Grain boundaries are sites where impurities and defects accumulate in the PVK layer, leading to thin film degradation.^5–7^ Larger-sized PVK grains result in films with reduced bulk and surface defects and improved protection against moisture.

Hybrid PVKs with mixed-halide structures present interesting performance for photovoltaic devices due to their optoelectronic properties.^8–10^ Specifically, different hybrid lead halide structures (APbX_3_) have produced efficient and stable PSCs.^11,12^ In this structure, A presents a mixture of cations, usually methylammonium (MA^+^), formamidinium (FA^+^), and Cesium (Cs^+^), while X is a combination of halide ions (I^–^, Br^–^, or Cl^–^). The perovskite thin film was prepared from a precursor solution by using the spin-coating technique. During this procedure, the PVK growth follows a heterogeneous nucleation process,^13,14^ where external sites (e.g., substrate) contribute to the nucleus formation and, subsequently, perovskite crystal growth. This procedure can be assisted by the supersaturation of the precursor solution that can be induced using the antisolvent method.^15,16^ In this method, a nonsolvent of the perovskite compounds is added to assist (or accelerate) the removal of the solvent used in the precursor solution during the spin-coating step.

Several procedures have been used to improve perovskite crystal growth. For instance, Gao et al.^17^ used CsPb_2_Br_5_ nanocrystals in the perovskite precursor solution to control crystal growth. This modification directly converts to a cubic α-phase, where the nanocrystals act as seeds to induce crystal growth. Bi et al.^18^ introduced poly­(methyl methacrylate) (PMMA) in the antisolvent, resulting in an improved crystallization process and larger grain sizes due to the heterogeneous nucleation assisted by the PMMA. Alexander et al.^19^ used a small molecule (Allantoin) in the perovskite precursor solution. This approach also improved the crystallization process by passivating defects and resulting in a perovskite film with larger grains. Recently, nanoparticles (NPs) have been used to improve the overall photovoltaic response of PSCs. These NPs can be used in different layers for multifunctional purposes. For instance, Gao et al.^20^ used CsPbBr_3_ nanoparticles in the antisolvent to enhance the nucleation step. In this approach, the CsPbBr_3_ nanoparticles act as nucleation sites and assist in crystal growth. In another approach, gold nanoparticles (Au NPs) were added to the perovskite precursor solution,^21^ resulting in larger crystal grains and reducing the number of defects. In this case, the authors showed that the Au NPs led to the lateral growth of monolithic grains. In a similar approach, ZnO nanoparticles were used in the PbI_2_ precursor solution; these NPs also act as additional nucleation sites.^22^

Besides the positive effect on structural features, metallic NPs can enhance the light absorbance and photocurrent by creating “hot spots” with localized electric fields in the interfaces or within the active layer. This arises from the localized surface plasmon resonance (LSPR) effect in these NPs.^23–26^ Mohammadi et al.^23,25^ showed theoretically the impact of the size and the distribution of Au NPs on the light absorbance and photovoltaic response of the MAPbI_3_ PVK layer. In another approach, the Au NPs and graphene composites have been deposited at the interface between the PEDOT: PSS and the perovskite layers.^26^ In this case, the authors observed an overall increase in the photovoltaic parameters, which was also attributed to the LSPR effect of the Au NPs.

To synthesize metallic nanoparticles (NPs) with controlled dimensions and uniform distribution, without the need for surface functionalization, laser ablation in organic solvents offers an effective method, particularly for photovoltaic and related applications.^27,28^ Herein, Au NPs were produced by the laser ablation in liquids method (LASis), while using anhydrous chlorobenzene (CB) as the liquid medium. Further, the Au NPs in the CB suspension were used in the antisolvent method to prepare CsFAMA PVK films. The resulting films were characterized using morphological, optical, and structural techniques and tested in PSCs devices. To our knowledge, this is the first report about using Au NPs prepared by LASis in CB and used as an antisolvent to produce a CsFAMA PVK film. The modified devices showed improvements in stability and hysteresis index with a superior PCE.

## 2 Experimental Section

### 2.1 Synthesis of Au NPs by LASiS

The Au NPs were prepared by using the LASIS method, which comprises a Q-switched Nd:YAG laser (Raycus RFL-P50QB, 1064 nm), operating at 50 kHz, delivering 150 ns pulses at the fundamental harmonic, and energy per pulse of 0.5 mJ. The laser beam was focused with a 20 cm lens on a gold target (Williams Advanced Materials, 5N), producing a 40-μm spot size. The target was placed 3 mm under anhydrous chlorobenzene (volume 15 mL). The irradiation time was 5 min, and the process was repeated two times under the same experimental conditions to yield a total colloidal volume of 30 mL. Then, the resulting suspension is harvested in a closed reagent flask.

The resulting colloidal Au NPs are stable over time because, during the laser ablation process, nanoparticles are often formed with a thin layer of adsorbed molecules (such as solvent molecules) or ions on their surfaces. Although chlorobenzene has a low dielectric constant, the availability of chloride ions as subproduct in the ablation solution results in a lower but sufficient surface charge,^29,30^ that ensures adequate electrostatic repulsion, eliminating the need for additional precursors or stabilizers to maintain colloidal stability until further processing.

### 2.2 Perovskites—Synthesis and Processing

The solvents used for the perovskite precursor solution Cs_0.05_FA_0.79_MA_0.16_Pb­(I_0.83_Br_0.17_)_3_ (CsFAMA) were dimethylformamide (DFM, Biotech. grade, ≥99.9%, Sigma-Aldrich) and dimethyl sulfoxide (DMSO, anhydrous, ≥99.9%, Sigma-Aldrich). All precursor solutions were prepared in a nitrogen atmosphere (1.4 M) from concentrations at 1.5 mol L^–1^ of PbI_2_ (99.9%, TCI AMERICA) and 1.5 mol L^–1^ of PbBr_2_ (≥99.9%, TCI or Puratronic), both in a 4:1 v/v mixture of DMF: DMSO, CsI (99.9%, Dyenamo) at the concentration of 1.5 mol L^–1^ in DMSO, and 1.2 mol L^–1^ of FAI (99.9%, Great Cell or Dyenamo) in a 4:1 v/v mixture of DMF: DMSO and MABr (>99.9%, Great Cell or Dyenamo) in a concentration of 1.3 mol L^–1^ in a 4:1 v/v mixture of DMF/DMSO. The perovskite thin films used for morphological characterization were prepared onto ITO substrates (Psiotec Ltd.), previously cleaned in an ultrasonic bath with acetone (5 min) and isopropanol (5 min), subsequently. Then, the substrates were dried with nitrogen and exposed to a plasma cleaner for 1 min. The precursor solution (50 μL) was spin-coated in two steps: 1000 rpm/10 s and 6000 rpm/20 s. The volume of 200 μL of CB antisolvent, or Au NPs suspended in CB antisolvent (200 μL), was dropped in the final 10 s of the second step. During this step, the Au NPs suspended in CB were used “as-synthesized” or diluted in CB in the 1:1, 1:2, 1:4, and 1:8 volume ratios. Further, the resulting films were annealed at 100 °C for 30 min.

### 2.3 Device Preparation

The solar cells produced in this work used the following geometry: glass/FTO/SnO_2_(30 nm)/KCl/CsFAMA (pure or Au NPs-modified, 500–600 nm)/Spiro-OMeTAD (171 nm)/Au (80 nm). The FTO patterned substrates were cleaned in an ultrasonic bath using aqueous Hellmanex (2% v/v) solution for 25 min and deionized water, acetone, and isopropyl alcohol (IPA) for 10 min each, followed by drying with a N_2_ flow. Using a UV-ozone chamber, the substrates were treated for 30 min. Then, the electron transport layer (ETL) was deposited by spin coating onto the FTO substrate using the tin­(IV) chloride pentahydrate (SnCl_4_·5H_2_O) in IPA (0.05 mol L^–1^) (60 μL) solution. The ETL layers were annealed at 180 °C for 1 h. Using spin coating, a potassium chloride (KCl) solution (10 mmol L^–1^ in water) (100 μL) was deposited on the ETL films and then annealed at 100 °C for 10 min. The FTO/SnO_2_ substrates were treated in a UV-ozone chamber for 30 min, and the CsFAMA solution was spin-coated as described above by using the antisolvent method. The hole transporting layer (HTL) was prepared as follows: 100 mg of Spiro-OMeTAD (70 mM) was dissolved in 1 mL of chlorobenzene and magnetically stirred at room temperature for 30 min. Then, the additives tBP (36 μL), 20 μL of LIFTSI solution (520 mg in 1 mL of ACN, 1.8 M), and 8 μL of FK209 solution (375.8 mg in 1 mL of ACN, 0.25 M) were added to this Spiro-OMeTAD solution and remained upon stirring for 10 min. The hole transport layer (HTL) solution of Spiro-OMeTAD (50 μL) was spin-coated onto the CsFAMA films. The samples were stored in the dark for 12 h for the oxidation process of the HTL, and then, the Au electrodes were deposited by thermal evaporation using a vacuum chamber (∼5 × 10^–6^ mbar), at a rate of 0.1 A s^–1^ (4 nm) and 1.0 A s^–1^ (66 nm).

### 2.4 Thin Film Characterization

#### 2.4.1 UV–Vis Spectroscopy

Ultraviolet–visible (UV–vis) absorbance spectra were acquired from Au NPs in CB suspension placed in a quartz cuvette and from Au NPs films deposited onto a quartz substrate.

Steady-state photoluminescence (PL) spectra measurements: PL spectra were measured in an Ocean Optics QEPro spectrofluorometer with a 365 nm LED directly on the surface of the substrate (Glass/PVK).

Time-Resolved Photoluminescence (TRPL): TRPL measurements were performed on a Horiba Jobin Yvon FL3–22-iHR-320 spectrofluorometer equipped with a time-correlated single-photon counting (TCSPC) system (FluoroHun-B) coupled to a 485 nm pulsed LED while monitoring the emission at λ_emi_ = 750 nm.

#### 2.4.2 Atomic Force Microscopy (AFM)

The Au NPs were processed as thin films onto glass substrates by spin coating. Approximately 100 μL of the Au NPs suspension was dropped onto the substrate in dynamic mode, with the substrate rotating at 3000 rpm during the drop. Then, the resulting Au NPs films were analyzed using an AFM microscope model SPM-9700 HT from Shimadzu, in tapping mode, and using a high-resolution AFM probe (SHR300, Budget Sensors, force constant 40 N/m, resonance frequency 300 kHz, gold overall coating, DLC spike not coated). The size distribution histogram was obtained by using the “particle analyses” resource available in the NanoMapping 3D software from Shimadzu.

#### 2.4.3 Dynamic Light Scattering (DLS)

DLS measurements were performed with a Microtrac Nanotrac Ultra size analyzer to obtain the hydrodynamic particle size distribution.

#### 2.4.4 Scanning Electron Microscopy (SEM)

SEM images were acquired using a JEOL JSM-6500F field emission scanning electron microscope with an acceleration voltage of 5 kV.

#### 2.4.5 Grazing Incidence Wide-Angle X-ray Scattering (GiWAXS)

GiWAXS measurements were performed at the ESRF beamline ID10,^31^ using photon energy *E* = 22.5 keV. A Dectris EIGER 4 M detector recorded the reciprocal space maps.

#### 2.4.6 X-ray Diffraction Measurements (XRD)

XRD data were collected by using a Shimadzu XRD-7000 diffractometer equipped with Cu Kα radiation (λ = 1.5418 Å). The measurements were performed by scanning the 2θ range from 5 to 80° with a step of 0.02° and an acquisition time of 0.4 s per step.

#### 2.4.7 Current–Voltage Characteristics

Density current versus voltage (*J*–*V*) curves under illumination (1 sun, 100 mW/cm^2^) of the PSCs were measured using a class A Solar Simulator (AM 1.5G, HAL-320, Asahi Spectra Co., Ltd.) and a Keithley 2400 (SourceMeter), in a voltage range from 0 to 1.2 V (forward scan and reverse scan) with steps of 10 mV and a delay time of 0.01 s. The simulator (in 1 sun) was calibrated by using a silicon reference solar cell with a KG5 filter. The active area of PSCs (area under illumination) was defined using a shadow mask (0.16 cm^2^) during the *J*–*V* measurement.

## 3 Results and Discussion

The versatility of the LASiS method enables the synthesis of nanosized metallic particles in various liquid media, including polar solvents such as water^32^ and isopropyl alcohol,^33^ as well as nonpolar organic solvents like chlorobenzene (CB).^28^ CB is particularly noteworthy due to its application as a nonsolvent in the preparation of PVK films. Initially, the optical and morphological properties of the synthesized Au nanoparticles (Au NPs) were characterized. Figure 1a shows the absorbance spectrum acquired from the Au NPs suspension, featuring a broad band at the wavelength region of ∼560 nm, corresponding to the LSPR band of nanosized Au particles. The size distribution, presented in Figure 1b, indicates particle sizes ranging from 2 to 38 nm. Topography and phase AFM images acquired from the Au NPs, Figure 1c,d, show that the particles are predominantly spherical with a minor presence of nanoplates. Complementary DLS analyses have been carried out (Figure S1), which also pointed out the presence of nanosized Au NPs suspended in the CB solvent.

**Figure 1:**
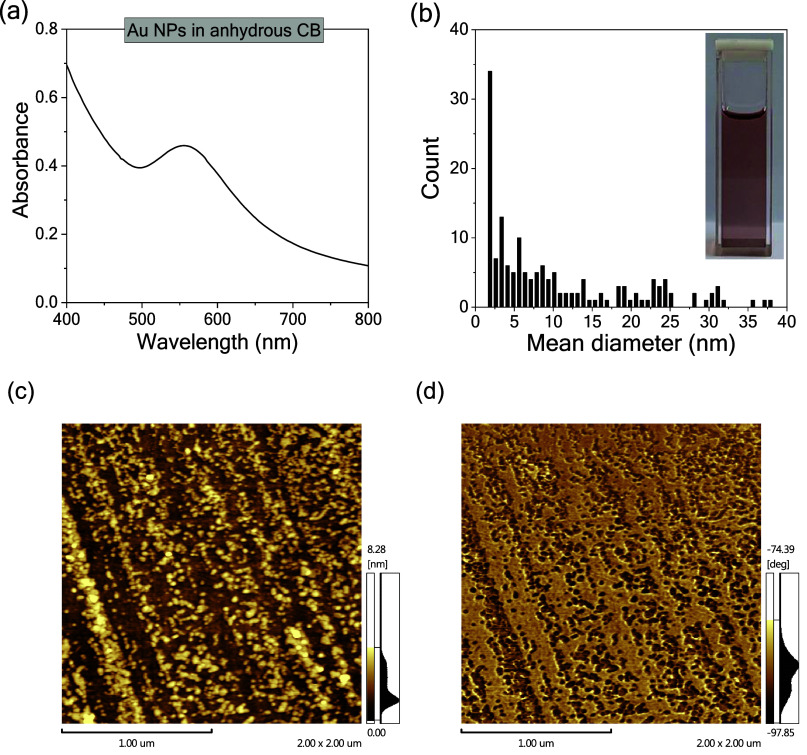
(a) UV–vis absorbance spectrum acquired from Au NPs in anhydrous CB, (b) diameter distribution measured from AFM images, (c) height, and (d) phase images of Au NPs films produced by spin coating in a dynamic method deposited on a glass substrate. Scale bars indicate 1 mm in the AFM images.

The concentration of Au NPs in CB can be consistently reproduced by maintaining the same LASiS synthesis conditions. In this study, the as-synthesized Au NPs suspension was diluted with CB (v/v), using ratios ranging from 1:1 to 1:8. Upon application of Au NPs-modified suspensions during the antisolvent step in the preparation of CsFAMA PVK films, significant morphological changes were observed. Figure 2 shows the SEM images, which indicate that the control film, prepared with pure CB as an antisolvent, exhibits grain sizes smaller than those of the Au NPs-modified PVK films. In general, larger grain size leads to fewer grain boundaries, enhances resistance to humidity,^5^ and reduces charge recombination rates in solar cells.^34^ The cross-section profile images revealed that Au NPs-modified CsFAMA films exhibit denser crystal packing and reduced film thickness compared with the control. This reduction in thickness may result from strain variations throughout the film, as suggested by the XRD results (Figure S2).

**Figure 2:**
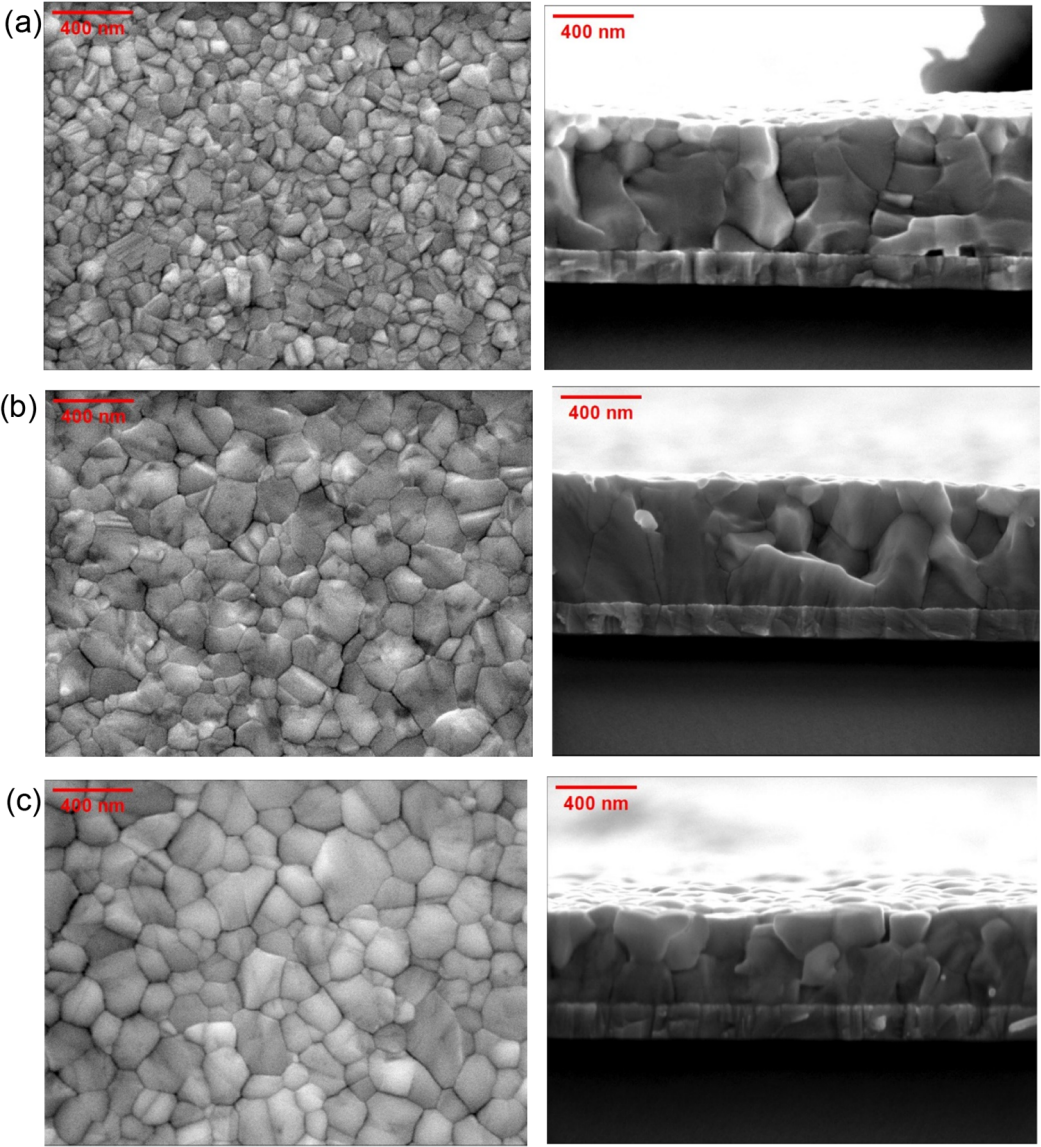
Surface and cross-sectional SEM images acquired from (a) control and Au-modified CsFAMA PVK films produced with (b) concentrated and (c) diluted (1:1 v/v %) Au NPs suspensions. Scale bars are 400 nm.

Both control and Au NPs-modified CsFAMA films (with varying NPs concentrations) were also characterized by AFM. Figure 3 shows the corresponding height and phase images. Consistent with the SEM images, these AFM images revealed that increasing the Au NPs concentration during the antisolvent step led to a larger grain size and increased surface roughness; the quantitative values are presented in Figure 4.

**Figure 3:**
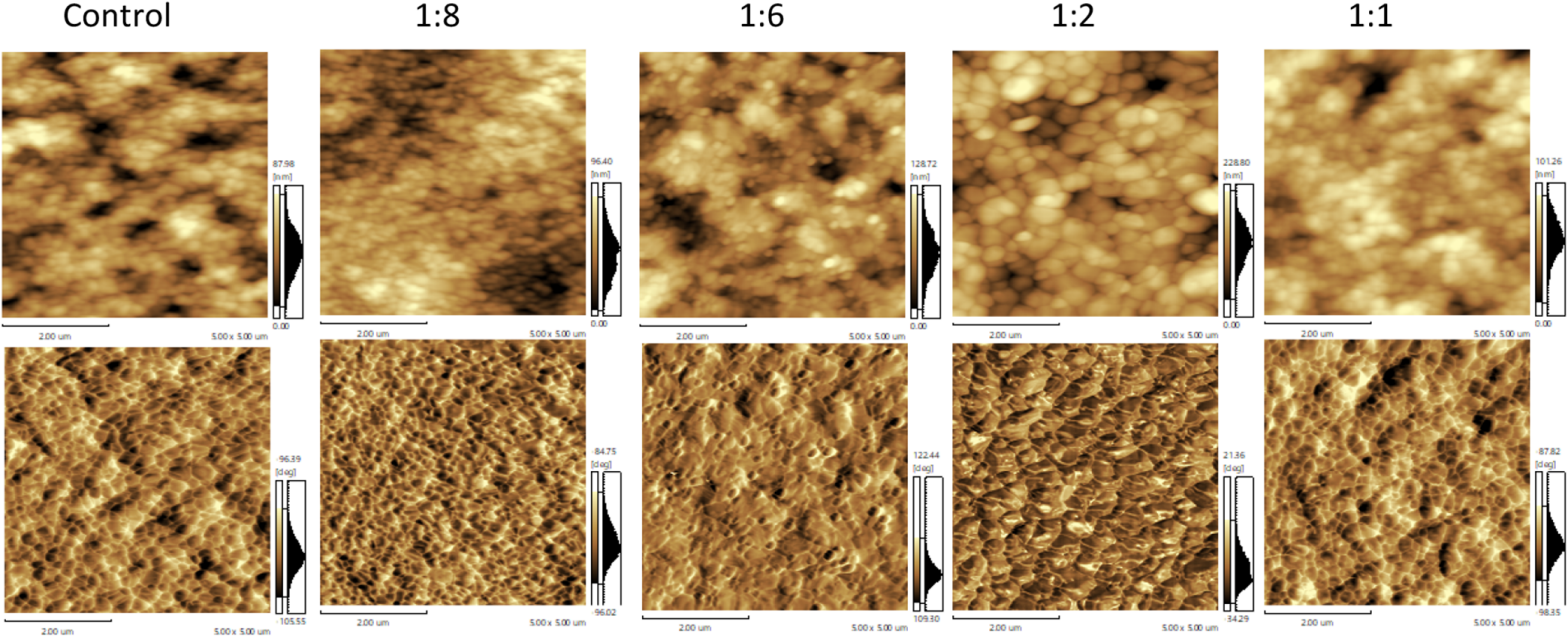
Height (top) and phase (bottom) images acquired from control and Au NPs-modified CsFAMA films. Scale bars indicate a wavelength of 200 nm.

**Figure 4:**
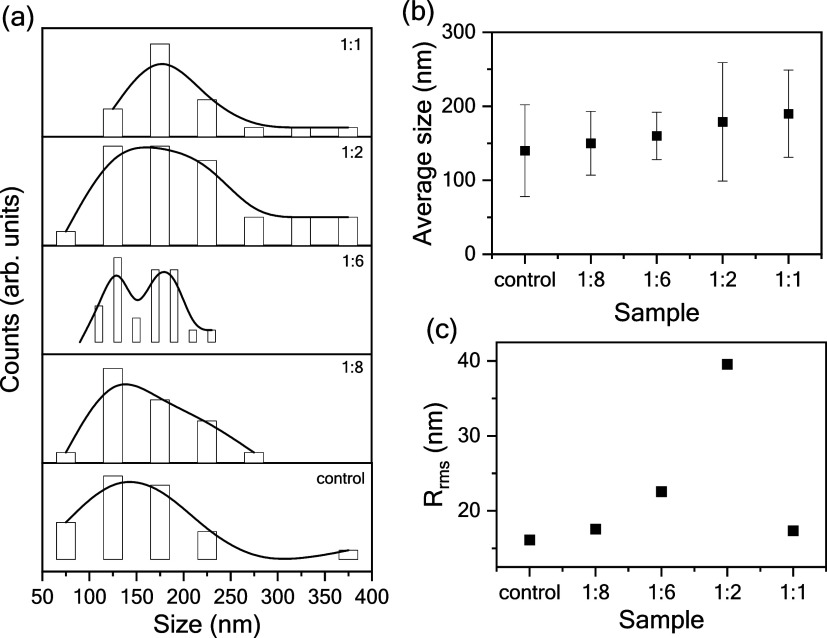
(a) Grain size distribution, (b) average size, and (c) surface roughness (*R*_rms_) acquired from control and Au NPs-modified CsFAMA films.

Analyses of the grain size distribution revealed that the addition of Au NPs broadened the distribution toward larger values and, in some cases, produced a bimodal distribution. Consequently, the average diameters were found to be 140 ± 62 and 190 ± 59 nm for the control and Au NPs (1:1)-modified CsFAMA films, respectively. Surface roughness increased with the Au NPs content, reaching a maximum *R*_rms_ of 39 nm in Au NPs (1:2)-modified CsFAMA films. Height AFM imaging indicated denser grain packing in Au NPs-modified films, evidenced by the reduced amount of holes along the surface when compared with the control film. Mainly, the phase AFM images also showed larger regions of enhanced crystallinity in the films containing Au NPs.

These morphological changes and enlarged grain sizes are attributed to NPs-assisted heterogeneous nucleation and subsequent grain growth. The heterogeneous nucleation process occurs at the interface or surfaces that act as favorable external nucleation sites.^13^ Consequently, Au NPs present in the antisolvent facilitate heterogeneous nucleation during spin-coating and solvent drying, as illustrated in Figure 5.

**Figure 5:**
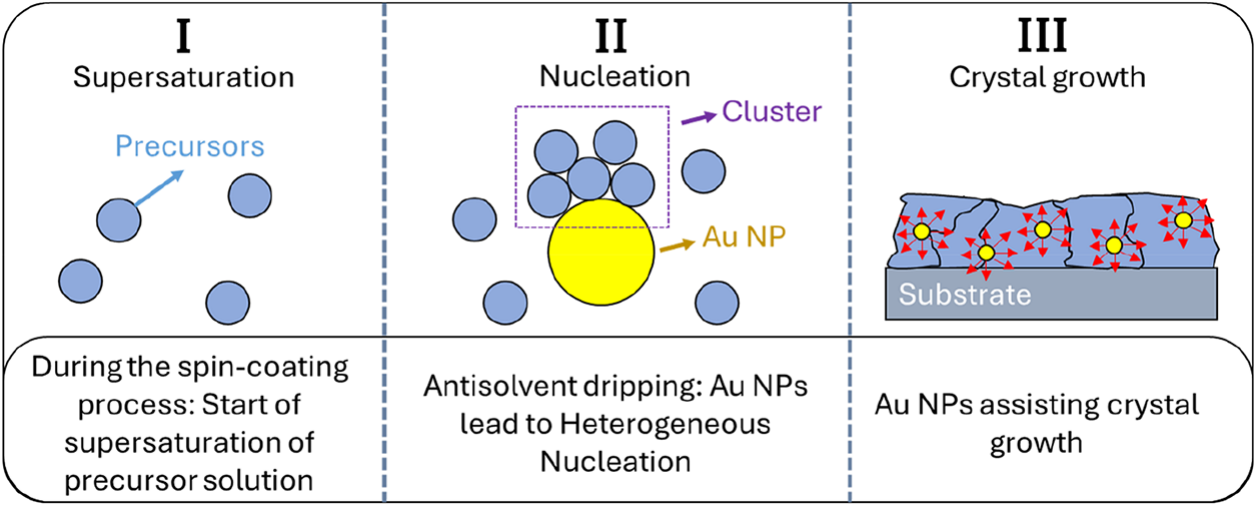
Representation of heterogeneous nucleation induced by Au NPs in the antisolvent step. The red arrows indicate the direction of grain growth.

During the deposition, the PVK precursor solution becomes supersaturated (stage I), after which Au NPs surfaces act as nucleation sites (stage II) and subsequently facilitate PVK crystal growth (stage III). For heterogeneous nucleation to proceed, the Gibbs free energy barrier at the interface between the nucleus and the Au NPs surfaces must be overcome.^14,35^ Under these conditions, the supersaturated PVK solution can adsorb onto Au NPs surfaces, providing favorable interfaces for nucleation. Crucially, the contact angle between the nucleus and the Au NPs surface must be low, implying proper wetting and a low interfacial energy. These features facilitate adsorption and reduce the nucleation barrier.

As demonstrated by Wang et al.,^36^ the so-called “seed engineering” strategy effectively promotes heterogeneous nucleation by lowering the nucleation energy barrier and enhancing the nucleation rate. In this study, the GIWAXS measurements reveal that incorporating Au NPs via the antisolvent step accelerates the nucleation and facilitates the conversion of intermediate phases into the photoactive α-phase. This may arise from the fact that Au NPs provide more nucleation sites. Intermediate phases formed during film deposition are known to degrade the quality of perovskite film and negatively affect the crystal formation.^37^ Therefore, suppression of these intermediate phases and enhancement of the photoactive α-phase are essential for achieving high-quality perovskite films. The heterogeneous nucleation process enables rapid consumption of precursors, which accelerates the growth of the photoactive α-phase while suppressing the formation of nonphotoactive phases, such as PbI_2_. As demonstrated by Bi et al.,^18^ the use of poly­(methyl methacrylate) (PMMA) during the antisolvent step enables a “seed engineering” strategy that controls nucleation and crystal growth. Specifically, PMMA interacts with PbI_2_ to form a PMMA-PbI_2_ adduct, which both accelerates heterogeneous nucleation and slows subsequent crystal growth. This dual mechanism leads to smoother films, improved grain size regularity, and overall enhanced crystallinity. These improvements result in perovskite films of superior electronic quality and enabled power conversion efficiencies of up to 21.6 %. In another approach, Qin et al.^38^ reported that the incorporation of core–shell Au@CdS nanospheres into the antisolvent triggered heterogeneous nucleation and enhanced crystal growth. The authors demonstrated that an intermediate Au@CdS-PbI_2_ phase forms preferentially at grain boundaries, promoting beneficial energy alignment between the perovskite layer and the hole transport layer. This strategy resulted in improved interfacial properties and overall device performance.

Compared to films prepared with a 1:1 Au NP suspension, CsFAMA films produced using more concentrated NP suspensions exhibited smaller grain sizes; see Figure 2b,c. This effect likely arises because a higher density of Au NPs promotes more nucleation events and intergrain interactions during crystal growth, thereby limiting average grain size. Consequently, there is a clear correlation between NPs concentration and crystal growth dynamics.^21^ These effects are corroborated by AFM analyses, which showed that an increasing Au NP concentration influenced the morphology, grain size, and surface roughness.

The resulting films were also analyzed by the XRD method. As shown in Figure S2, the XRD patterns of films prepared under the same batch and procedures confirm that the Au NPs incorporation does not affect bulk purity. In both control and Au NPs-modified films, all peaks correspond to the cubic α-phase of CsFAMA. However, significant changes in lattice parameters are observed. A slight shift to higher 2θ values, for instance, the (211) peak, appears at 31.85° in the control film and at 31.80° in the Au NPs-modified film, a feature that indicates a decrease in interplanar spacing and lattice contraction. This shift is attributed to strain induced by the Au NPs during film formation.^39^ The strain values have been calculated from XRD data using the de Williamson–Hall method.^40^ Negative strain values were obtained from control (ε = −0.00216), 1:1 (ε = −0.01337), and 1:8 (ε = −9.07897 × 10^–4^) samples, indicating a compressive strain. Similar structural effects have been reported for perovskite films modified with Au NPs.^41,42^ Regarding residual PbI_2_, the relative intensity of the (100) diffraction peak (at approximately 2θ = 12.70°) is lower in Au NPs-modified films compared to the control film prepared under identical conditions. Moreover, this reduction correlates with the concentration of Au NPs; for instance, the films prepared using more diluted Au NPs suspensions contain less unreacted PbI_2_, in proper accordance with the GIWAXS measurements.

Moreover, the effect of Au NPs was also analyzed by UV–vis spectroscopy. As shown in Figure 6a, perovskite films prepared with highly diluted Au NPs suspension (v/v ratios of 1:4 and 1:8) exhibited increased absorbance in the 400–500 nm wavelength range. This enhancement likely arises from localized plasmonic “hot spots” both in the bulk and at the surface of the perovskite film, which amplify photon absorption. Similar behavior has been observed in PVK films deposited onto Au NPs-modified graphene interlayer.^43^ In contrast, films with higher Au NPs loadings showed reduced absorbance. This may correlate with increased residual Pb_I2_ and the formation of nonphotoactive hexagonal phases; both features contribute to reducing photon absorption. Significant changes were observed in the emission spectrum in Figure 6b. The CsFAMA film produced with a higher Au NPs concentration in the antisolvent exhibited increased emission intensity, spectral broadening, and a red shift. The intensity enhancement is likely due to LSPR-mediated emission amplification, though this effect did not result in improved device performance.^44^ Despite the LSPR band of Au NPs being at a higher energy than the emission arising from perovskite, there is a minor spectral overlap that enables surface plasmon–exciton coupling.

**Figure 6:**
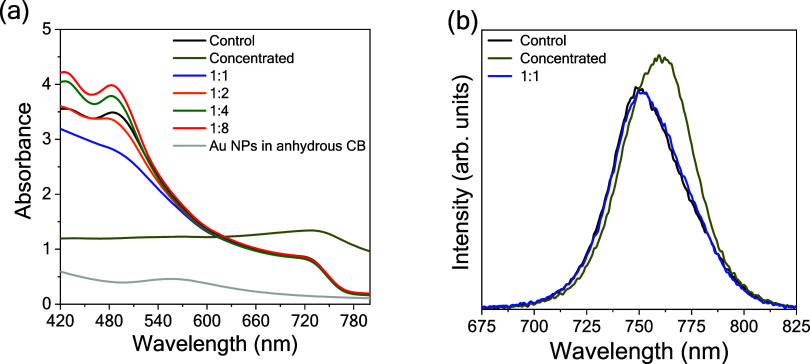
(a) UV–vis absorbance and (b) steady-state emission spectra acquired from control and Au NPs-modified CsFAMA PVK films.

Concerning the decay times, while monitoring the emission band at ∼750 nm, the results pointed out that the CsFAMA film presented a two-component decay curve with decay times of 79 and 245 ns, which are in good accordance with the literature.^45^ Comparatively, the decay times acquired from Au NPs-modified CsFAMA presented longer decay times when prepared with concentrated Au NPs suspension, which may indicate a higher order in these films, Table S1 and Figure S3. However, the films prepared with diluted Au NPs suspensions presented reduced values for τ_1_ decay time and higher A_2_ contribution in TRPL, indicating more efficient charge extraction and reduced nonradiative recombination.

The crystallization process was monitored in situ using GIWAXS measurements to evaluate it in real time. Figure 7 shows the monitoring during both the spin-coating and annealing steps for the control and Au NPs-modified PVK films. All the peaks were indexed according to the literature.^11^ During the spin-coating step, initially, two peaks are observed in *q* = 2.14, 2.46 Å^–1^, which can be assigned to the ITO substrate. At *t* = 30 s, the antisolvent is added. The results pointed out that, in the sample produced with Au NPs diluted suspension (1:1 v/v), there is a faster appearance of the peaks at *q* = 0.99, 2, and 3 Å^–1^, which are assigned as cubic α-phases of PVK (see the horizontal orange bars in Figure 7).

**Figure 7:**
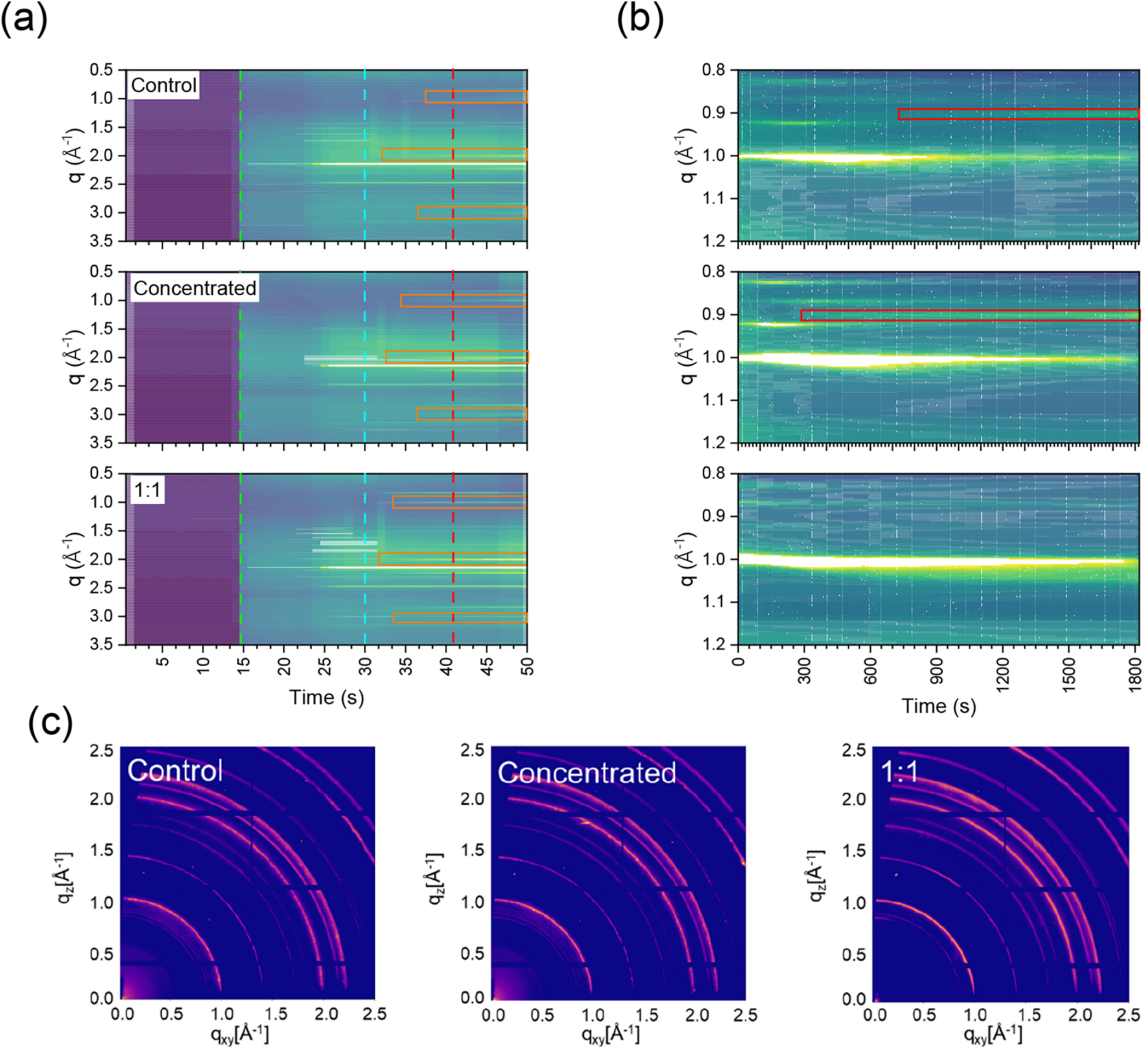
In situ GIWAXS data acquired (a) during the spin coating and antisolvent steps (green, blue, and red dashed lines indicate the start of spin-coating, antisolvent dropping, and final spin-coating, respectively; orange bars indicate cubic (α) peaks at *q* = 0.99, 2, and 3 Å^–1^), (b) during the thermal annealing step (red bars indicate the PbI_2_ peak at *q* = 0.9 Å^–1^), and (c) reciprocal space maps from CsFAMA PVK films produced as control (without Au NPs), concentrated (Au NPs suspension as synthesized), and diluted (Au NPs suspension: CB 1:1 v/v %).

Concerning the thermal annealing step, Figure 7b, the Bragg peaks located at *q* = 0.82 Å^–1^ and *q* = 0.87 Å^–1^ are assigned as hexagonal 4H phase and 6H phase,^11,46^ respectively. Other works also reported the emergence of complex hexagonal phases during the crystallization process.^47^ The feature correlated with residual PbI_2_ at the scattering vector at *q* = 0.9 Å^–1^ emerges after 280 s in the concentrated sample and after 583 s in the control sample with a higher intensity. Surprisingly, this feature has not been observed in the PVK film produced using the Au NPs diluted sample (1:1 v/v %) during the thermal annealing and in the measurements in fresh PVK films (Figure 7c). According to the literature, the presence of PbI_2_ has both benefits and disadvantages. For instance, Zhang et al.^48^ showed that the excess of PbI_2_ localized at grain boundaries and interfaces with charge transport layers can passivate defects, improving the efficiency of PSCs. On the other hand, Akbulatov et al.^49^ point out that PbI_2_ in excess decreases the performance of PSCs due to the photolysis effect when exposed to temperatures >55 °C. Therefore, the excess of PbI_2_ can improve performance with a passivation effect but sacrifice stability during operating time.^50^ Thus, since the Au NPs-modified PVK films present a lower amount of PbI_2_, it is expected that the PSC devices may present an improvement in stability during operation time.

Moreover, the molar phase fractions between the amount of residual PbI_2_, PVK cubic, and PVK hexagonal phases were evaluated using different incidence angles. In this work, the incidence angles of 0.15 and 0.3° were used for surface-sensitive (Figure 8a) and bulk-sensitive (Figure 8b) analysis, respectively. The absence of the peak at *q* = 0.9 Å^–1^ (PbI_2_) in the PVK film produced with the diluted Au NPs suspension has also been confirmed in bulk-sensitive and surface-sensitive analysis. PbI_2_ fraction is more significant for the PVK control film along the surface and bulk. Comparatively, PVK films produced with Au NPs present more hexagonal (nonperovskite) phase fractions along the bulk than the PVK control film. However, the PVK film produced with diluted Au NPs suspension presents a more cubic phase fraction than other samples along the surface. Specifically, the 6H hexagonal polymorph phase (*q* = 0.87 Å^–1^) is slightly more evident for samples with Au NPs, with the 1:1 sample presenting less hexagonal phase fraction on the surface and more in the bulk than the concentrated sample. The control PVK film presents more 4H hexagonal polymorph phase (*q* = 0.82 Å^–1^) in the surface and bulk regions than PVK films with Au NPs. Hexagonal phases present a face-sharing octahedra structure.^11,51^ Thus, its presence in the PVK film diminishes the charge transport compared with the corner-sharing cubic structure, decreasing the overall device performance. Therefore, this is also an important effect of the Au NPs, improving the amount of cubic phase at the interface with the hole transporting layer.

**Figure 8:**
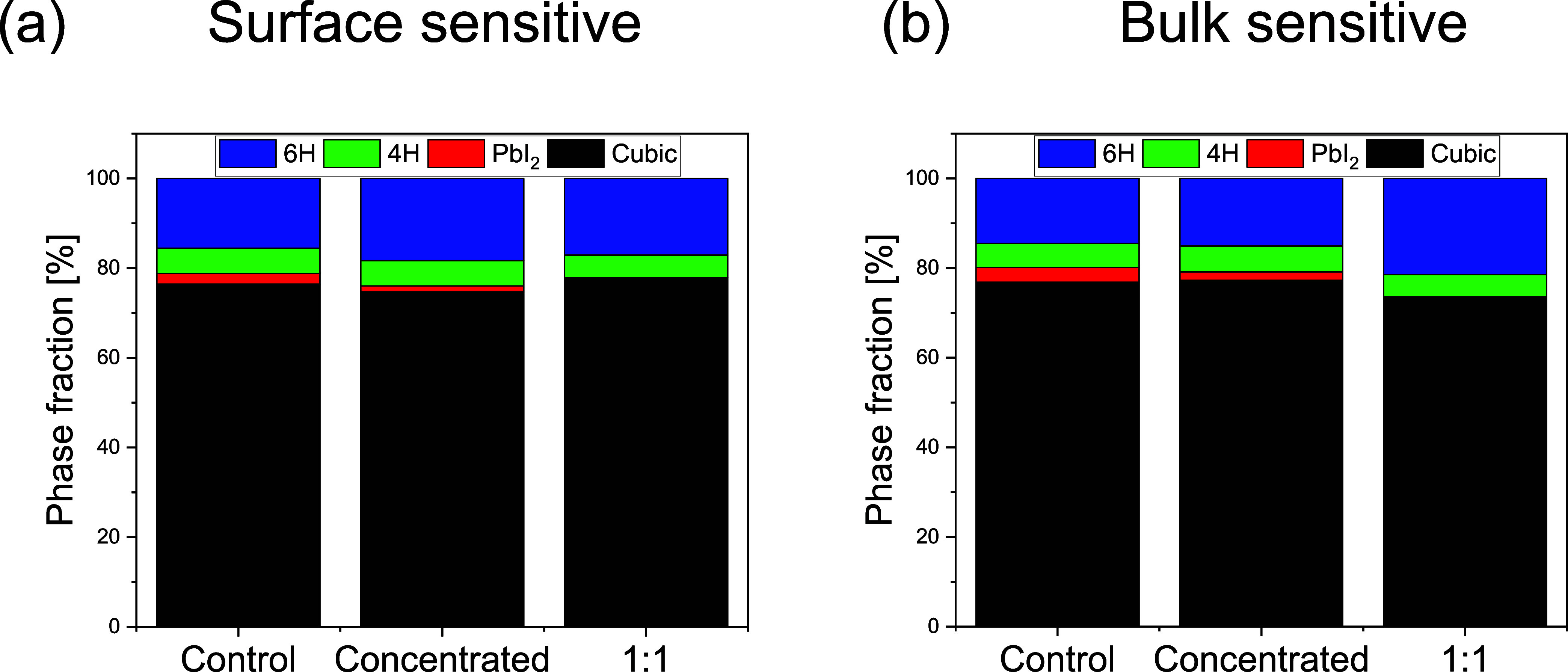
Phase fraction calculated for PbI_2_, cubic, and hexagonal phases from peak integration of GIWAXS data measured with different incidence angles: (a) surface-sensitive (α = 0.15°) and (b) bulk-sensitive (α = 0.3°).

As seen in the GIWAXS measurements, the crystallization process was improved by the faster nucleation and a lower amount of the PbI_2_ phase in the Au NPs-modified PVK films.

The PSCs (glass/FTO/SnO_2_/KCl/PVK/Spiro-OMeTAD/Au) with a Au NPs-modified CsFAMA PVK layer were fabricated to evaluate the effects on device performance. The photovoltaic parameters acquired for the PSCs are displayed in Figure 9 and Table S2. Positive results on the open-circuit voltage (*V*_oc_), short-circuit density current (*J*_sc_), fill factor (FF), and power conversion efficiency (PCE) were observed for devices produced with low content of Au NPs. The effect of the nanoparticle concentration on the series (*R*_s_) and shunt (*R*_sh_) resistances was also evaluated. The results indicated that devices produced with a concentrated Au NPs suspension presented a reduced *R*_sh_; this low *R*_sh_ leads to higher leakage current, which reduces *V*_oc_ and FF. The highest *R*_sh_ was observed in the optimized device produced at the concentration of 1:8 (v/v). These results are also included in Table S2.

**Figure 9:**
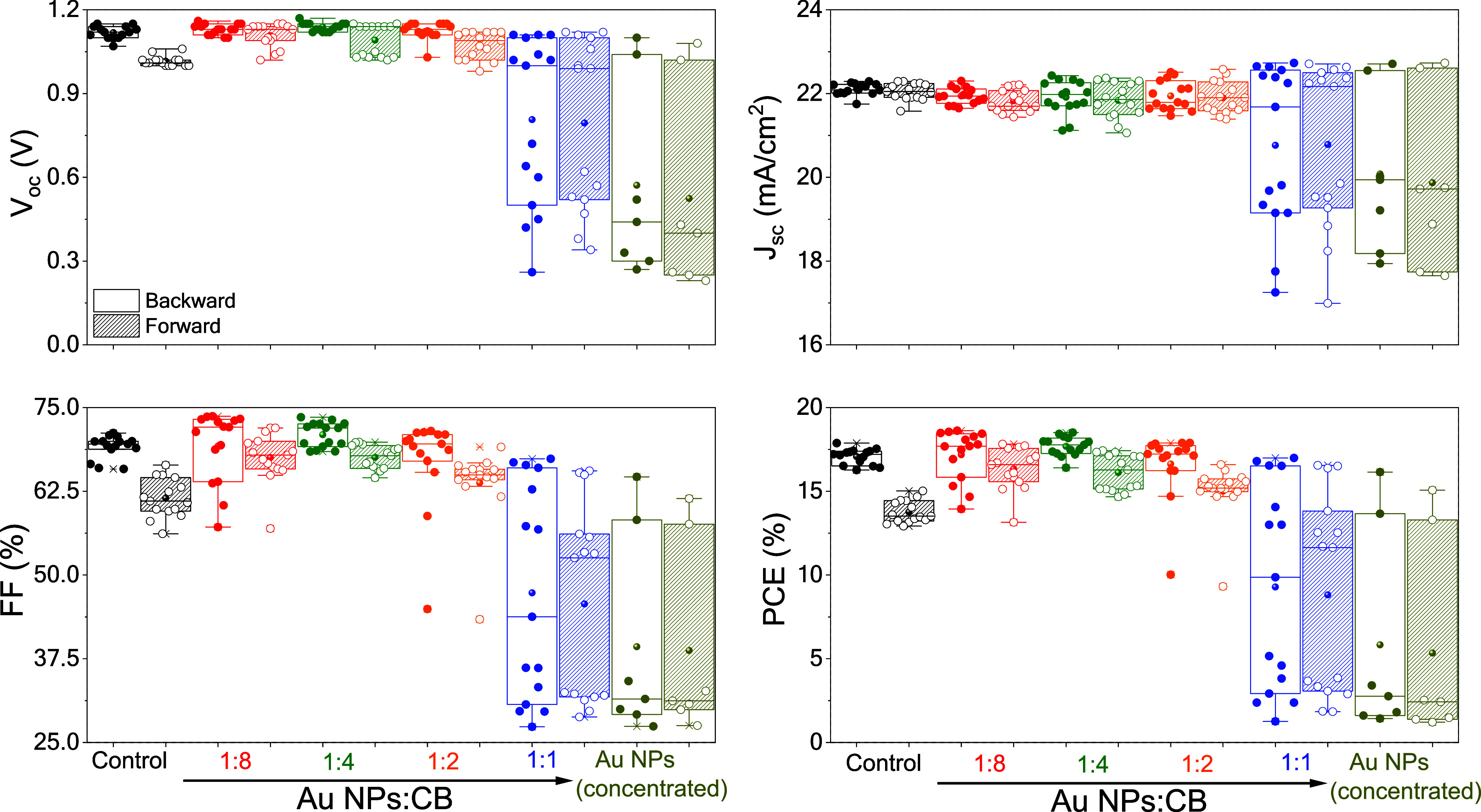
Forward and backward photovoltaic parameters acquired from glass/FTO/SnO_2_/KCl/CsFAMA:AuNPs/Spiro-OMeTAD/Au devices. The control device was produced using pure CsFAMA.

Devices produced with modified PVK having Au NPs with a concentration higher than 1:2 (v/v) presented worse performance with a larger spread in the photovoltaic parameters. These results may indicate that the excess of Au NPs can cause a worsening in charge transport properties at the bulk or at interfaces, as indicated by the lower *V*_oc_ value. Moreover, as shown in Figure 6, the Au NPs-modified CsFAMA films with a higher amount of nanoparticles presented reduced light absorbance, which impacts the photovoltaic response. Due to the poor photovoltaic response of these devices (concentrated and 1:1), the efforts were focused on monitoring the performance of devices modified with lower concentrations of Au NPs. The best photovoltaic stability was achieved with the Au NPs-modified CsFAMA layer produced with diluted Au NPs suspension (1:8 v/v), which resulted in a *V*_oc_ of 1.15 V, *J*_sc_ of 21.96 mAcm^–2^, FF of 73.70%, and PCE of 18.62% (backward) in the first measurement, as seen in Figure 10b. These values are superior to those acquired from the control device in backward and forward acquisition modes (Figure 10a). Moreover, by monitoring the devices over a time of up to 1000 h, the Au NPs-modified CsFAMA PSCs kept superior photovoltaic performance over time, indicative of the positive impact of the larger grain sizes induced by the Au NPs-assisted heterogeneous nucleation during the antisolvent step. The data about monitoring devices over 1000 h is presented in Figure S4. The stability test of PSCs was performed using the International Summit on Organic Photovoltaic Stability (ISOS) protocol, specifically ISOS-D1 (samples were kept in the dark and under ambient air, without encapsulation).^52^

**Figure 10:**
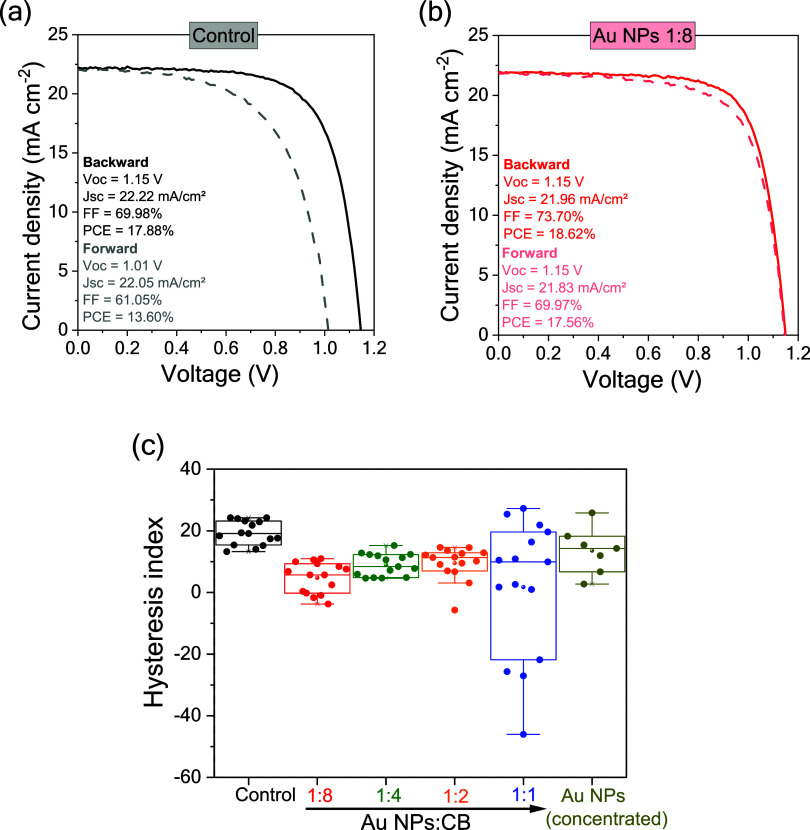
*J–V* curves (forward and backward scans) under 1 sun illumination acquired from (a) control and (b) optimized glass/FTO/SnO_2_/CsFAMA:AuNPs­(1:8 v/v)/Spiro-OMeTAD/Au devices. Hysteresis index data (c) for glass/FTO/SnO_2_/KCl/CsFAMA:AuNPs­(v/v)/Spiro-OMeTAD/Au devices. The control devices were produced using pure CsFAMA.

Another feature observed in the modified devices with Au NPs is the reduction of the hysteresis index HI = (PCE_backward_ – PCE_forward_)/PCE_backward_,^53^ shown in Figure 10c. As presented in Figure 10b, the Au NPs-modified device (1:8 v/v) exhibits a PCE of 18.62% when using the scan direction from short-current to forward bias and a minor decrease to 17.56% when using the opposite scan direction. In comparison, the control device presents a difference of about 16–13% in PCE under different scan directions. Similar results in terms of reduction in the hysteresis index have also been observed for devices prepared with diluted concentrations of Au NPs, as presented in Figure 9. This reduction in hysteresis index may be correlated with balanced charge accumulation at the interfaces.^54^ As indicated by the phase fraction results, the Au NPs-modified CsFAMA PVK films have essential structural changes that impact the charge transport features, for instance, lower concentration of PbI_2_ and the top surface region composed mainly of the cubic phase of PVK.

Therefore, analysis of the hysteresis index, photovoltaic parameters, and TRPL decay revealed that lower Au NP concentrations (1:8 and 1:4) led to reduced hysteresis indices, improved device performance, shorter *t*_1_ values, and higher A_2_ contributions in TRPL, indicating more efficient charge extraction and reduced nonradiative recombination. Furthermore, maximum power point (MPP) tracking confirmed this behavior. Compared to the control (*J*–*V* PCE = 17.88%, MPP PCE = 14.71%), the Au NP-modified device (1:8) exhibited only a minor drop in performance (*J*–*V* PCE = 18.62%, MPP PCE = 17.61%) (Figure S5).

For the intermediate Au NPs concentration (1:2), although the τ_1_ value remained short and A_2_ contribution was high, device performance began to decline, possibly due to nanoparticles aggregation or nonuniform and irregular interfaces. In the 1:1 Au NP condition, some devices exhibited negative hysteresis along with a longer τ_1_ value and lower A_2_ contribution, suggesting delayed charge extraction and asymmetric interfacial dynamics. The concentrated Au NPs-modified CsFAMA film showed poor photovoltaic performance, likely due to inefficient charge extraction or charge accumulation. These results indicate that while Au NPs can enhance interfacial properties and charge dynamics at low concentrations, excessive loading may introduce interfacial barriers or charge-blocking regions, resulting in delayed carrier recombination and asymmetric charge redistribution.^55–57^

## 4 Conclusions

The implementation of the LASiS method for synthesizing Au NPs in chlorobenzene antisolvent has proven to be highly effective in enhancing the morphological and structural quality of CsFAMA perovskite films. Introducing Au NPs during the antisolvent step promotes heterogeneous nucleation and grain enlargement, yielding films with improved crystallinity, reduced thickness, more uniform density, and reduced formation of intermediate phases and PbI_2_ residues. In terms of optical properties, an optimal Au NP concentration was identified up to a 1:8 (v/v) dilution, resulting in enhanced light absorption (likely via plasmonic hot spots) and shortened carrier lifetimes when compared with the control device, as demonstrated by emission and decay times measurements. In situ GIWAXS analysis further confirmed a faster crystallization process, favoring the photoactive α-phase and supporting an improved photovoltaic performance. Moreover, the optimized Au NPs-modified devices exhibit a reduced hysteresis index. Since hysteresis can be caused by capacitive effects, an improvement in the hysteresis index may indicate improvements in charge transport features, which can be caused by improvements in morphology induced by Au NPs that act as seeds during the crystallization process.

## Supplementary Material



## Notes

DLS measurement (Figure S1); XRD patterns (Figure S2); TRPL decay curves (Figure S3); stability test of PSCs (Figure S4); maximum power point (MPP) (Figure S5); decay times (Table S1); and device parameters (Table S2) (PDF)
