# Supplementary material for: Effects of Au Nanoparticles Suspended in Chlorobenzene Antisolvent on Mixed-Halide Perovskites
Source: ACS Omega. 2025 Sep 16;10(38):44298–310. doi: 10.1021/acsomega.5c05967 (PMC12489622; doi:10.1021/acsomega.5c05967)
Supplement: Supplementary file 1 [file ao5c05967_si_001.pdf]

Supporting Information file

# Effects of Au nanoparticles suspended in chlorobenzene antisolvent on mixed-halide perovskites

# Effects of Au nanoparticles suspended in chlorobenzene antisolvent on mixed-halide perovskites

Eduardo H. dos Santos Rosa[1,4], Andreia de Morais[2], Francineide Lopes de Araújo[3], Paul Zimmermann[4], Alexander Hinderhofer[4], Jilian Nei de Freitas[2], Rafael Eleodoro de Góes[5], Arandi Ginane Bezerra Jr[5], Andreia Gerniski Macedo[5,*], Wilson José da Silva[1,5], Ana Flávia Nogueira[3], Frank Schreiber[4]

*[1]CPGEI, Department of Electronics, Universidade Tecnológica Federal do Paraná, 80230-901 Curitiba, PR, Brazil*

*[2]CTI Renato Archer, 13069-901 Campinas, SP, Brazil*

*[3]Laboratório de Nanotecnologia e Energia Solar (LNES), Institute of Chemistry, Universidade Estadual de Campinas (UNICAMP), 13083-970 Campinas, SP, Brazil*

*[4] Institute for Applied Physics, University of Tübingen, 72074 Tübingen, Germany*

*[5]PPGFA, Department of Physics, Universidade Tecnológica Federal do Paraná, 80230-901 Curitiba, PR, Brazil*

**e-mail: agmacedo@utfpr.edu.br*

*Contents:*

Figure S1 – Dynamic light scattering (DLS) measurement of Au NPs in CB.

Figure S2 – (a) XRD patterns and (b) partial XRD patterns at the region of (211) diffraction peak acquired from control and Au NPs-modified CsFAMA films deposited on Glass/FTO/$SnO_2$ substrate.

Figure S3– TRPL decay curves acquired from control and Au NPs-modified CsFAMA films. a) control, b) concentrated, c) 1:1 (v/v), d) 1:2 (v/v), e) 1:4 (v/v), f) 1:8 (v/v).

Figure S4 – Stability test of PSCs (control and Au NPs 1:8) conducted following the ISOS-1D protocol: (a) PCE and (b) standardized PCE as a function of air exposure time.

Figure S5 – Maximum power point (MPP) tracking under continuous illumination for the (a) control and (b) Au NPs-modified device (1:8).

Table S1 – Decay times acquired from control and Au NPs-modified CsFAMA films.

Table S2 – Photovoltaic parameters of all PSCs (3 batches) were measured under illumination (100 mW/cm$^2$) in the backward scan (B) and forward scan (F). Average and standard deviation values were obtained based on 15 devices. For devices based on CsFAMA:AuNPs (concentrated),

statistical analysis was based on 7 devices. The values in parentheses are for the best-performing PSCs.

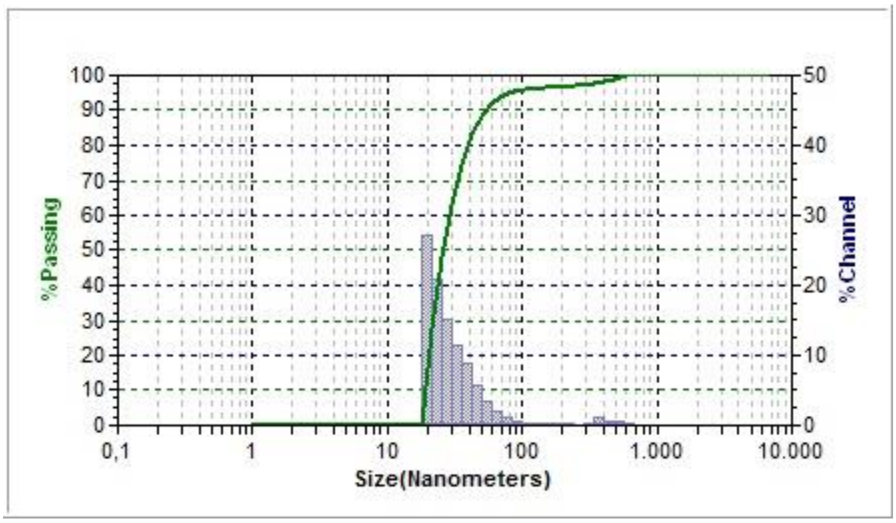


Figure S1 – Dynamic light scattering (DLS) measurement of Au NPs in CB.

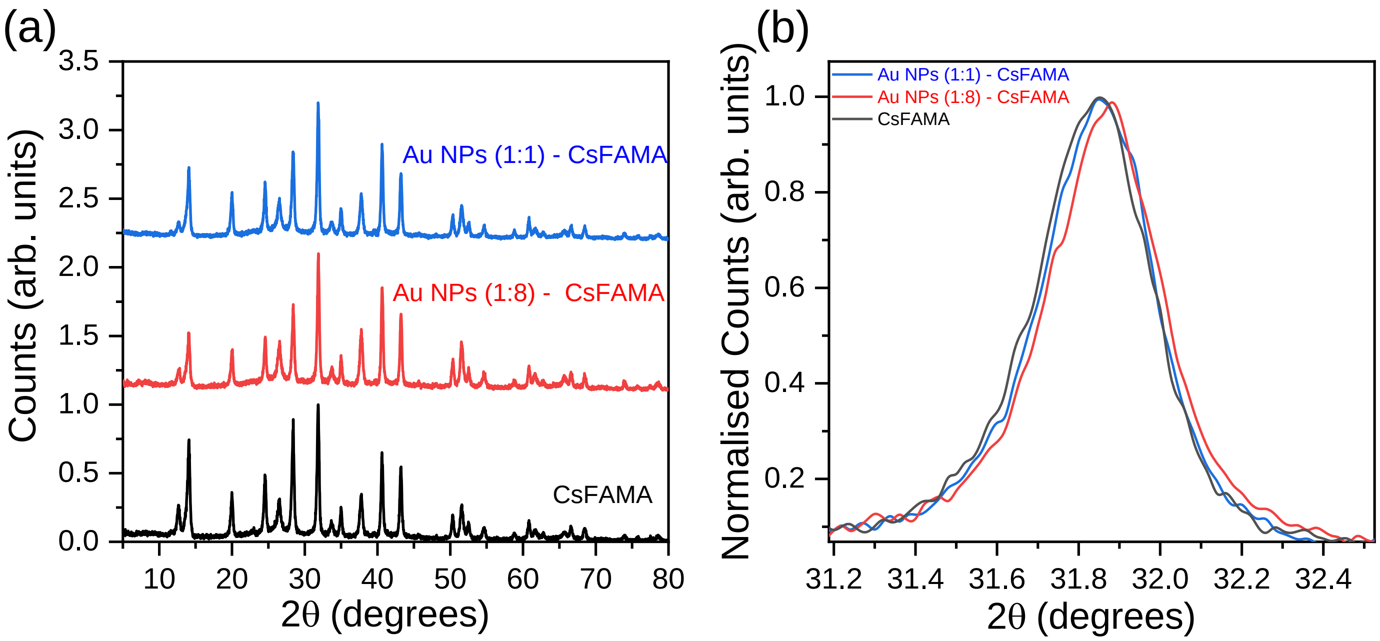


Figure S2 – (a) XRD patterns and (b) partial XRD patterns at the region of (211) diffraction peak acquired from control and Au NPs-modified CsFAMA films deposited on Glass/FTO/$SnO_2$ substrate.

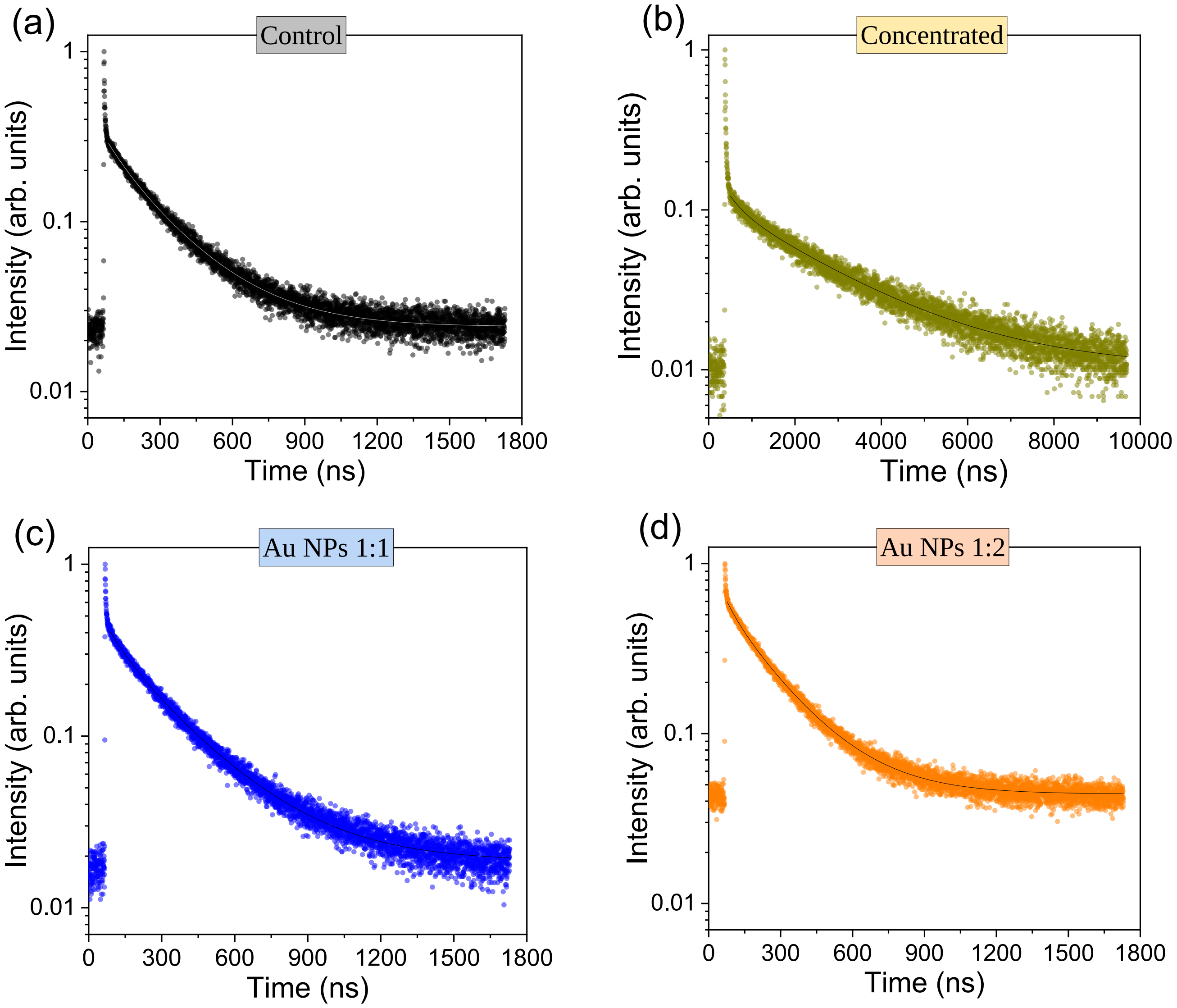

(a)
Control
Intensity (arb. units)
1
0.1
0.01
0
300
600
900
1200
1500
1800
Time (ns)
(b)
Concentrated
Intensity (arb. units)
1
0.1
0.01
0
2000
4000
6000
8000
10000
Time (ns)
(c)
Au NPs 1:1
Intensity (arb. units)
1
0.1
0.01
0
300
600
900
1200
1500
1800
Time (ns)
(d)
Au NPs 1:2
Intensity (arb. units)
1
0.1
0.01
0
300
600
900
1200
1500
1800
Time (ns)

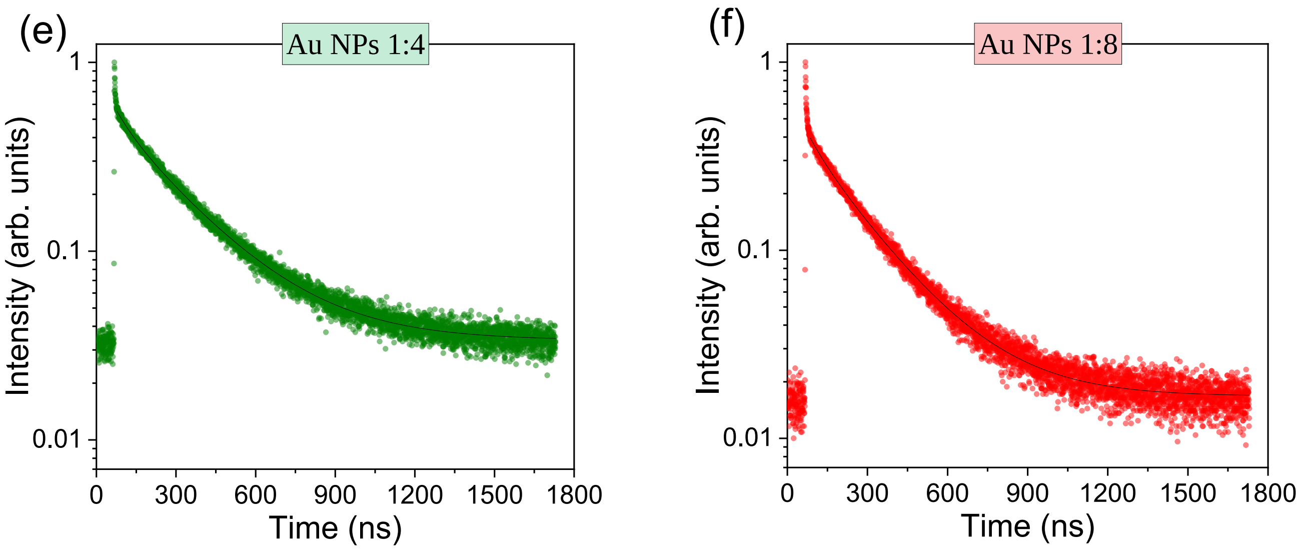


Figure S3– TRPL decay curves acquired from control and Au NPs-modified CsFAMA films. a) control, b) concentrated, c) 1:1 (v/v), d) 1:2 (v/v), e) 1:4 (v/v), f) 1:8 (v/v).

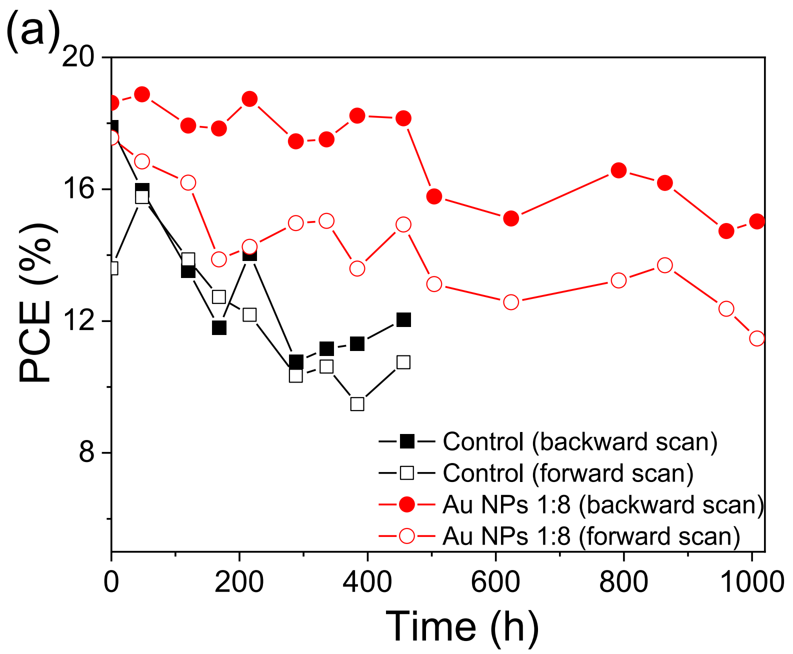

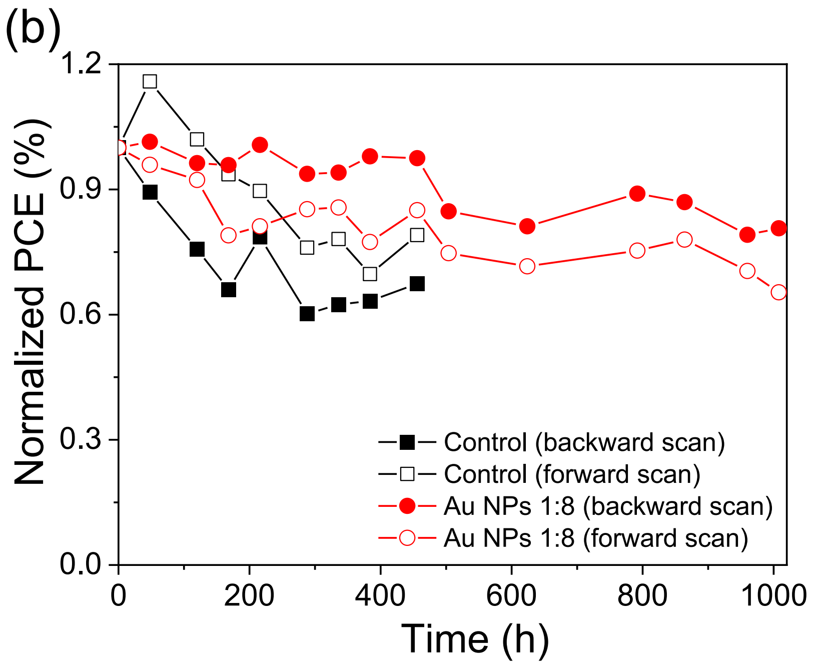


Figure S4 – Stability test of PSCs (control and Au NPs 1:8) conducted following the ISOS-1D protocol: (a) PCE and (b) standardized PCE as a function of air exposure time.

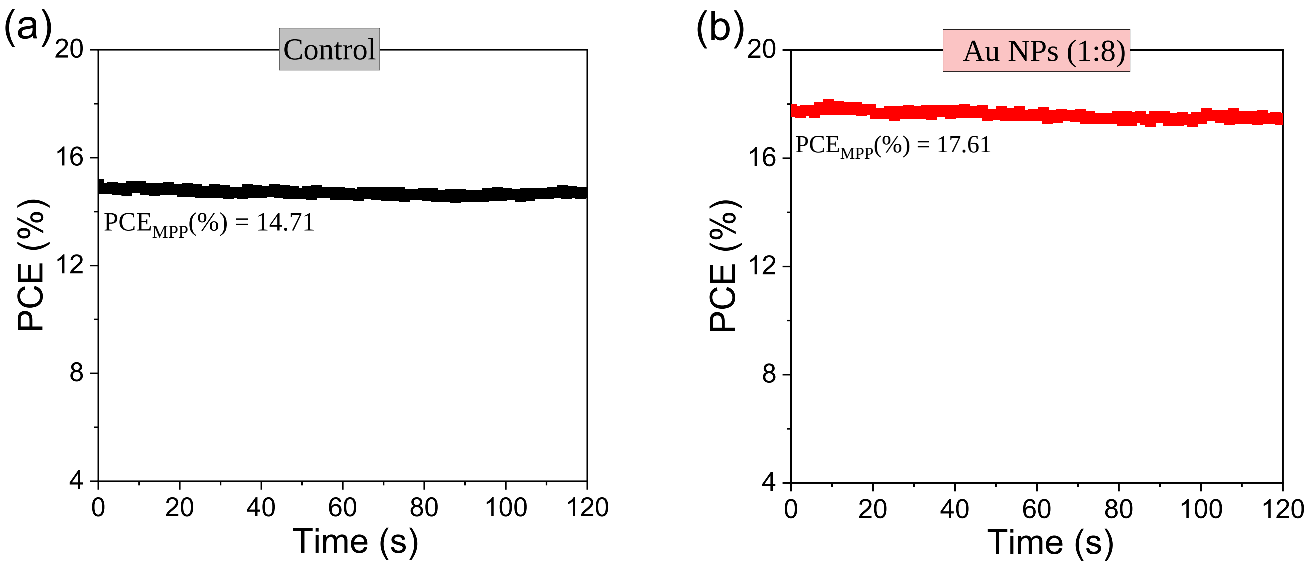


Figure S5 – Maximum power point (MPP) tracking under continuous illumination for the (a) control and (b) Au NPs-modified device (1:8).

**Table S1** – Decay times acquired from control and Au NPs-modified CsFAMA films.

| **Sample** | **$T_1$ (ns)** | **$A_1$** | **$T_2$ (ns)** | **$A_2$** |
|---|---|---|---|---|
| control | 79.31981 | 0.15721 | 245.1938 | 0.30157 |
| Au NPs 1:8 | 59.04463 | 0.23695 | 223.3889 | 0.47552 |
| Au NPs 1:4 | 57.55116 | 0.07556 | 259.8304 | 0.42081 |
| Au NPs 1:2 | 52.45754 | 0.08865 | 216.2673 | 0.45504 |

| | | | | |
|---|---|---|---|---|
| Au NPs 1:1 | 88.01476 | 0.09868 | 281.1558 | 0.29244 |
| Au NPs concentrated | 337.5570 | 0.02261 | 2365.514 | 0.08886 |

**Table S2** – Photovoltaic parameters of all PSCs (3 batches) were measured under illumination (100 mW/cm$^2$) in the backward scan (B) and forward scan (F). Average and standard deviation values were obtained based on 15 devices. For devices based on CsFAMA:AuNPs (concentrated), statistical analysis was based on 7 devices. The values in parentheses are for the best-performing PSCs.

| Devices | scans | $V_{oc}$ (V) | $J_{sc}$ (mA/cm$^2$) | FF (%) | PCE (%) | Rs (Ω cm$^2$) | Rsh (Ω cm$^2$) |
|---|---|---|---|---|---|---|---|
| CsFAMA (control) | B | 1.12 ± 0.02 (1.15) | 22.11 ± 0.14 (22.22) | 69.12 ± 1.69 (69.98) | 17.07 ± 0.47 (17.88) | 2.27 | 620 |
| | F | 1.02 ± 0.02 (1.01) | 22.05 ± 0.20 (22.05) | 61.48 ± 2.91 (61.05) | 13.77 ± 0.66 (13.60) | 2.14 | 200 |
| CsFAMA:AuNPs (1:8) | B | 1.13 ± 0.02 (1.15) | 21.94 ± 0.19 (21.96) | 69.25 ± 5.37 (73.70) | 17.19 ± 1.52 (18.62) | 2.21 | 1000 |
| | F | 1.11 ± 0.04 (1.15) | 21.83 ± 0.26 (21.83) | 67.54 ± 3.73 (69.97) | 16.33 ± 1.26 (17.56) | 2.33 | 520 |
| CsFAMA:AuNPs (1:4) | B | 1.14 ± 0.02 (1.17) | 21.91 ± 0.40 (21.78) | 70.95 ± 1.88 (72.62) | 17.69 ± 0.58 (18.51) | 2.45 | 840 |
| | F | | 21.84 ± 0.43 (21.55) | 67.58 ± 1.75 (68.86) | 16.12 ± 1.07 (17.07) | 2.92 | 470 |

| | | | | | | | |
|---|---|---|---|---|---|---|---|
| | | 1.09 ± 0.06 (1.15) | | | | | |
| CsFAMA:AuNPs (1:2) | B | 1.13 ± 0.03 (1.14) | 21.94 ± 0.35 (22.12) | 67.20 ± 6.97 (70.97) | 16.63 ± 2.01 (17.89) | 2.13 | 610 |
| | F | 1.07 ± 0.05 (1.12) | 21.90 ± 0.38 (21.90) | 63.75 ± 5.87 (66.34) | 15.00 ± 1.66 (16.27) | 2.58 | 350 |
| CsFAMA:AuNPs (1:1) | B | 0.81 ± 0.30 (1.11) | 20.76 ± 1.95 (22.73) | 47.34 ± 16.22 (67.35) | 9.29 ± 6.21 (16.99) | 5.64 | 700 |
| | F | 0.79 ± 0.31 (1.12) | 20.78 ± 1.95 (22.63) | 45.67 ± 14.65 (65.28) | 8.81 ± 5.92 (16.55) | 5.45 | 400 |
| CsFAMA:AuNPs (concentrated) | B | 0.57 ± 0.35 (1.10) | 20.08 ± 1.91 (22.71) | 39.30 ± 15.38 (64.66) | 5.83 ± 6.28 (16.15) | 2.14 | 210 |
| | F | 0.52 ± 0.37 (1.08) | 19.87 ± 2.09 (22.73) | 38.73 ± 14.32 (61.41) | 5.34 ± 6.08 (15.07) | 2.26 | 170 |
